# Variants and expression changes in PPAR-encoding genes display no significant association with schizophrenia

**DOI:** 10.1042/BSR20201083

**Published:** 2020-07-21

**Authors:** Xinrong Li, Yue Zhu, Maria Keaton, Ancha Baranova, Sha Liu, Xiaodong Hu, Qi Li, Long Cheng, Peng Zhou, Hongbao Cao, Yong Xu

**Affiliations:** 1 Department of Psychiatry, First Hospital /First Clinical Medical College of Shanxi Medical University, Taiyuan, China; 2 Department of Biomedical Engineering, Tianjin University, Tianjin, China; 3 School of Systems Biology, George Mason University (GMU), Fairfax, VA 22030, U.S.A.; 4 Research Center for Medical Genetics, Moscow, Russia

**Keywords:** bioinformatics, peroxisome proliferator-activated receptors, psychotic disorders

## Abstract

A few studies suggested the contribution of PPARs to the etiology of schizophrenia (SCZ). However, it is still not clear whether variants in PPAR-encoding genes have a direct association with SCZ. The potential linkage between SCZ and the variants within PPAR encoding genes (*PPARA, PPARD*, and *PPARG*) was tested in a large cohort genome-wide association study (GWAS). Then, a mega-analysis was conducted using 14 gene expression profiling experiments in various human brain regions. Finally, the expression levels of the three PPAR-encoding genes were quantified in early-onset SCZ patients. Only one *PPARG* polymorphisms, rs62242085, presented a minor frequency deviation in the SCZ cohort (*P*-value = 0.035). None of the PPAR-encoding genes presented significant expression change within the brain regions profiled in 14 datasets acquired from different populations (*P*-value > 0.14) or in the whole blood of early-onset overall SCZ patients (*P*-value > 0.22). However, compared with healthy female controls, female early-onset SCZ patients presented a moderate but significant decrease in the expression level of *PPARD* (LFC = −0.55; *P*-value = 0.02) and a strong, but non-significant decrease in expression of *PPARG* (LFC = −1.30; *P*-value = 0.13). Our results do not support a significant association between variants in PPAR-encoding genes and SCZ, but suggest a necessity to explore the role of *PPARD* and *PPARG* in early SCZ phenotypes, specifically in females.

## Introduction

Schizophrenia (SCZ) is a mental illness with high heritability, characterized by abnormal behavior and a decreased ability to integrate into reality [1]. As a psychiatric disorder, SCZ affects approximately 0.3–0.7% of people during their lifetimes [1] and imposes a heavy financial burden on families and society all over the world [2]. In 2016, SCZ contributed to about 13.4 million patients who lived with disability (YLD), equivalent to 1.7% of total YLDs globally [2]. The development of SCZ is influenced by multiple factors, including genetic, environmental, and life course events [3–5].

In recent years, several studies tested the association between peroxisome proliferator-activated receptors (PPARs) and SCZ, with inconsistent conclusions [6–12]. PPARs are a group of nuclear receptor proteins composed of PPAR-α (encoded by gene *PPARA*), PPAR-δ (by *PPARD*), and PPAR-γ (by *PPARG*); these proteins play important roles in the differentiation, development, and metabolism of mammalian cells [13]. PPARs regulate the transportation and expression of various genes [14], which harbor variants contributing to hundreds of diseases, including different types of cancer [15,16], metabolic disorders [6], and multiple mental health-related conditions, such as depression [17], Parkinson’s disease [18], anxiety [19], and brain dysfunction [20]. Given the obvious importance of PPARs in core metabolic processes and in WNT signaling, both in the brain and in peripheral tissues, a relatively weak connection between variants in PPAR encoding genes and psychiatric phenotypes is surprising.

On the one hand, some studies suggested the potential association between PPARs and the etiology of SCZ or its related clinical symptoms. For instance, Malan-Müller et al.’s work suggested that the product of *PPARG* affects metabolic syndrome in schizophrenia patients through the regulation of Apolipoprotein E (ApoE) [6]. Liu et al. reported that variants in *PPARG* may be associated with altered glucose levels and the course of disease in SCZ patients treated with antipsychotics [7]. Furthermore, a recent analysis of single-nucleotide polymorphisms (SNPs) within *PPARG* locus in 756 individuals undergoing an 18 month trial of 5 different antipsychotic medications revealed a correlation of these variants with an extent of weight gain [21]. In addition, the PPAR-α L162V polymorphism has been linked to nicotine dependence among SCZ patients [9]. *PPARA* gene has also been suggested to have a contribution to the etiology of SCZ in the Chinese Han population [8]. So far, no associations of *PPARD* variants and psychiatric conditions have been reported, except one family-based bipolar disorder study that pinpointed the importance of the Wnt pathway in general, and intronic *PPARD* variant rs2267665 in particular, in the development of this condition [22]. However, multiple other studies produced no support for the allelic association between PPARs and SCZ [10,23,24], even if a particular focus on the genetic variance of PPARs in SCZ was selected.

On the other hand, some studies have concluded that PPAR-encoding genes may not be associated with SCZ [10–12]. For example, Mathur et al. showed that SCZ in the British population was not associated with *PPARG* allelic variations [10]. Sun et al. also concluded that the *PPARD* gene might not be involved in the negative symptoms of SCZ in the Chinese population [12].

To address the above issues, we designed the present study to explore the PPAR–SCZ association at three different levels: polymorphism of PPAR encoding genes, their expression levels within particular brain regions, and expression levels in peripheral blood mononuclear cells (PBMCs) collected from treatment-naïve patients with early-onset schizophrenia, in a hope to uncover new insights regarding the roles of the PPAR group in the pathological development of SCZ.

## Materials and methods

The present study is organized in the following order. First, in a large cohort Genome-wide association study (GWAS), we examined associations of the polymorphic variants in all three PPAR-encoding genes, *PPARA, PPARD*, and *PPARG*. Then, for each of these genes, multiple gene expression profiles were acquired from human brain regions, and a mega-analysis was conducted. Finally, whole blood expression profiles of early-onset SCZ were interrogated to extract the expression levels of the three PPARs. The details were described as follows.

### Study of the variants of PPAR-encoding genes in SCZ patients

Variation in PPAR encoding genes was investigated in a large cohort Genome-wide association study (GWAS) dataset available from the Psychiatric Genomics Consortium (PGC) (https://www.med.unc.edu/pgc/results-and-downloads/), the largest GWAS study on SCZ made available so far [25]. A combined analysis of two large-scale GWAS datasets, which contains 9,444,230 variables, was performed. Dataset one includes 34,241 SCZ cases and 45,604 healthy controls from 49 ancestry-matched lineages (46 European and 3 East Asian lineages). The second dataset contains 3,241 SCZ cases, 45,604 healthy controls, and 1,235 affected offspring of a parent with SCZ. For both of the studies, the genotypes were processed by PGC using a unified quality control procedure [25].

Genomic sequences of PPAR-encoding gene loci were acquired from GeneCards (https://www.genecards.org). The location of *PPARA* is from 46594280 to 46631277 on chromosome 22, *PPARD* is from 35342558 to 35428191 on chromosome 6, and *PPARG* is from 12287485 to 12434356 on chromosome 3. The variants previously mapped to PPAR-encoding genes were matched to the GWAS results, and then the statistics describing the SCZ/control comparison were extracted.

### Mega-analysis using 14 expression data from brain samples

To explore the brain-specific levels of PPAR-encoding mRNAs, a mega-analysis of expression was conducted in 14 SCZ-related expression datasets (Table 1). For unbiased selection of datasets, the Illumina Correlation Engine (http://www.illumina.com) was searched with keyword ‘schizophrenia’. All datasets are publicly available at GEO. The data selection criteria were as follows: (1) The organism is Homo sapiens; (2) The data type is RNA expression by array; (3) The sample size is no less than 25 specimens; (4) The study has case–control design; (5) The dataset and its format files are publically available; (6) Specimens represent various regions of the brain. From each dataset, expression data for the normal controls and for SCZ patients were extracted and then re-analyzed. To note, we used the term ‘Mega-analysis’ instead of ‘meta-analysis’ in a reflection of the fact that the expression fold changes and comparison *P*-values of PPARs were derived from original data, rather utilizing results reported in previous studies.

**Table 1:** Key descriptors of 14 schizophrenia-related RNA expression datasets were selected for the present study

	*N* of Cases/Controls	Specimen studied	Population
**GSE12649**	35/34	Prefrontal cortex	Japan
**GSE12654**	13/15	Prefrontal cortex	Japan
**GSE12679**	16/11	Dorsolateral prefrontal cortex	United Kingdom
**GSE17612**	28/23	BA10	United Kingdom
**GSE21138**	30/29	Prefrontal cortex	U.S.A.
**GSE21935**	23/19	BA22	United Kingdom
**GSE26927**	10/55	Multiple Brodmann areas	United Kingdom
**GSE35974**	44/50	Parietal cortex	China
**GSE35977**	51/50	Parietal cortex	China
**GSE35978**	95/100	Parietal cortex	China
**GSE53987**	48/55	Prefrontal cortex (BA46)	U.S.A.
**GSE62191**	29/30	Frontal cortex	Brazil
**GSE87610**	65/72	Prefrontal cortex	U.S.A.
**GSE93987**	67/106	Dorsolateral prefrontal cortex	U.S.A.

The expression data were normalized and log2-transformed, if necessary. For each expression dataset and each gene, the log fold change (LFC) was calculated, then used as the index of effect size in mega-analysis. To explore the effect size of the expression levels of PPAR-encoding genes on SCZ phenotypes, both the fixed-effect model (FEM) and the random-effects model (REM) [26] were employed. Results from both models were reported and compared. To ascertain the variance within and between different studies, the heterogeneity of the mega-analysis was analyzed. If the total variance *Q* was equal to or smaller than the expected between-study variance (d*f*), the statistic ISq = 100% x (*Q* − d*f*)/*Q* was set as 0, and a fixed-effect model was selected for the mega-analysis. Otherwise, a random-effects model was selected. The *Q–P* represents the probability that the total variance is explained by within-study variance only. All procedures were executed in an individually-developed MATLAB (R2017a) mega-analysis package.

A multiple linear regression analysis was employed to uncover the possible influence of sample size, population region, and study date on the gene expression alterations detected in patients with schizophrenia. *P*-values and 95% confidence interval (CI) were reported for each of the factors. The analysis was done in Matlab (R 2017a) with the ‘regress’ statistical analysis package.

### Acquisition of PPAR expression profiles from PBMC samples

For each of three PPAR encoding genes, the levels of their expression were measured in total preparation of PBMCs from 19 individuals with early-onset schizophrenia (EOS), and 18 demographically matched controls (HC). A power estimation was conducted using the ‘sampsizepwr’ function in Matlab (version R2017a). We assumed that the mean and the standard deviation of the LFC of the whole genome were 0 and 0.5, respectively. To differentiate a significant varied gene with LFC ≥ 1 or LFC ≤ -1, the sample size should be ≥5 for each group involved in the comparison. Therefore, the sample sizes of the PBMC expression profiles analyzed here do satisfy the statistical power requirement.

All participants were unrelated Han Chinese recruited from North China and under the age of 18 years old. For each patient, EOS diagnosis was established independently by two physicians, in accord with the Diagnostic and Statistical Manual of Mental Disorders: Fourth Edition (DSM-IV) and the Chinese version of the Modified Structured Clinical Interview for DSM-IV, patient version (SCID-I/P). For each patient, the total PANSS score was ≥60, and IQ score ≥70. Patients with the following characteristics were excluded: (1) having an organic disease of heart, liver, and kidney; (2) having an immune disease and brain injury or congenital brain malformation, tumor of brain and epilepsy; (3) those with mental retardation and taking antipsychotic drugs, anti-manic drugs, antidepressants or mood stabilizers. In addition, we excluded patients with serious excitement or impulsive behavior.

The healthy controls (HC) were enrolled by matching EOS cohort in age and sex, and the criterion of never taken any drugs in the latest 30 days. Exclusion criteria were (1) did not meet the standards of include or exclude terms of SCZ patients; (2) a family history of any psychiatric or nervous system disease; (3) a history of a head injury or any inborn disease; (4) a history of hyperpyretic convulsion; and (5) being adopted or lives in a single-parent family. For all teenage participants, informed consent was signed by both parents. The present study was performed under the protocols approved by the First Hospital of Shanxi Medical University. One-way ANOVA was conducted to compare the expression levels of mRNAs of interest, with log fold change (LFC) and *P*-value reported for each comparison. Differentially expressed genes (DEGs) were recognized as LFC≥1 or ≤-1 and *P*-value<0.05.

Total RNA was extracted from all of the samples snap-frozen using TRIzol reagent (Invitrogen, Carlsbad, CA, U.S.A.), according to the manufacturer’s protocol [27]. Total RNA preps were quantified by the NanoDrop ND-1000, and RNA integrity was assessed by standard denaturing agarose gel electrophoresis as described before [27]

Agilent Feature Extraction software (version 11.0.1.1) was used to analyze acquired array images. Quantile normalization and subsequent data processing were performed using the GeneSpring GX v11.5.1 software package (Agilent Technologies).

## Results

### Variants in PPAR-encoding loci

**Figure 1:**
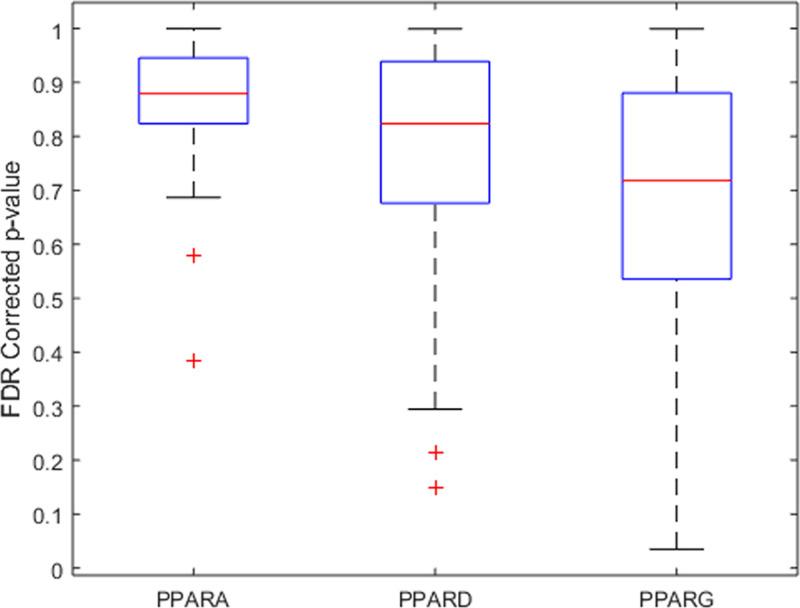
Boxplot of the FDR corrected *P*-values for the SNPs located within each PPAR-encoding locus The original *P*-values of the SNPs were extracted from the combined analysis of two large-scale GWAS data [18].

Variants located within PPAR-encoding loci were identified, and the SCZ/control comparison *P*-values and FDR corrected *q*-values were extracted (Figure 1). To understand the relative significance of these SNPs in the original study context, the GWAS results were ranked by *P*-values in ascending order, and the indexes of the PPARs SNPs were summarized, as shown in Table 2. Our results showed that none of the SNPs located in PPAR-encoding loci displayed any significant association with SCZ. For the detailed presentation of the results, please refer to Supplementary Table S1.

**Table 2:** PPARs genome-wide data by base pairs

Gene symbol	Number of respective SNPs	*P*-value	*q*-value	Rank by *P*-value	Rank by *P*-value (%)
*PPARA*	77	>0.036	>0.39	>1872585	>9.00%
*PPARD*	278	>0.0046	>0.15	>290658	>3.00%
*PPARG*	423	>0.00040	>0.035	>99041	>0.60%

Note: ‘Number of respective SNPs’ reflects only variants present in the GWAS data, among 9,444,230 SNPs in total; ‘*q*-value’ was the FDR corrected *P*-value; ‘Rank by *P*-value’ is the rank index by *P*-value in ascending order; ‘Rank by *P*-value (%)’ is ‘Rank by *P*-value’ divided by 9,444,230, which was the total number of SNPs assayed.

### The expression of PPARs-encoding mRNAs in brain regions

**Figure 2:**
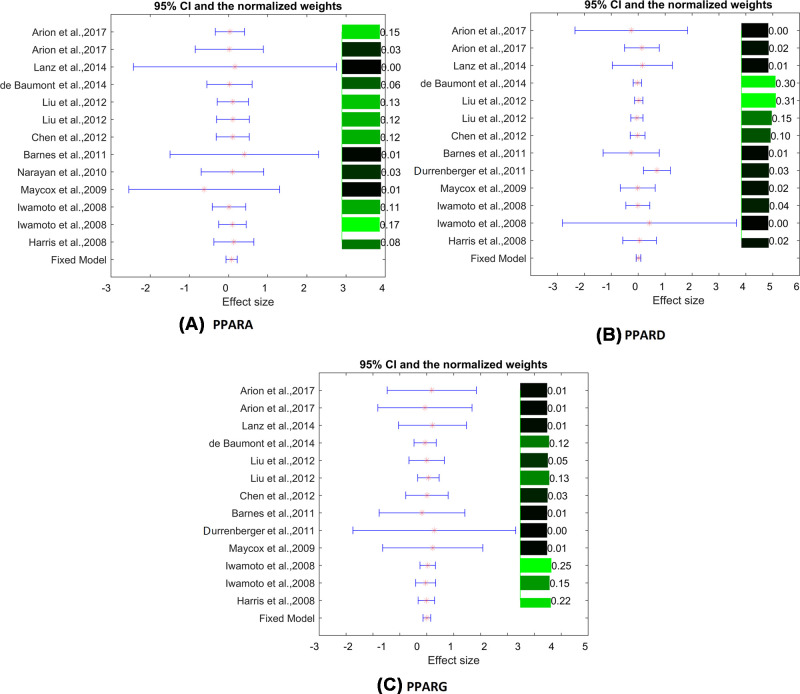
The effect size, 95% confidence interval, and weights for expression levels of genes encoding (A) PPAR-α; (B) PPAR-δ; (C) PPAR-γ The bar plot on the right of each figure represents the normalized weights for each dataset/study, ranged within (0, 1); the brighter (green) the color, the larger the weight (labeled right next to the bar). The star (in red) and lines (in blue) on the left are the mean of effect size (log fold change) and 95% confidence interval (CI) of each dataset/study, respectively.

Heterogeneity analysis showed non-significant between-study variance for all the three PPAR encoding genes analyzed (ISq = 0, *P*-value–*Q* > 0.90). Thus, a fixed-effect model (FEM) was selected for the analysis (Table 3). The effect sizes and related statistics of the mega-analysis of expression results are presented in Table 3. None of the three genes of interest showed statistical significance in this analysis (*P*-value>0.14; abs(LFC)<0.08). MLR results indicate that the mega-analysis results were not influenced by sample size, population region (country), and study age. The effect size, 95% confidence interval, and weights of each study are presented in Figure 2.

**Table 3:** The levels of PPAR encoding gene mRNAs in various brain regions do not contribute to schizophrenia

Gene Name	Mega-analysis results					MLR results			
	# of Study	LFC	*P*-value	ISq(%)	*P*-value–Q	Sample size	Country	Study age	Sample source
*PPARA*	13	0.080	0.14	0	1.00	0.92	0.023	0.75	0.0015
*PPARD*	13	0.012	0.39	0	0.63	0.99	0.82	0.52	2.73E-05
*PPARG*	13	0.0067	0.46	0	1.00	0.96	0.62	0.62	2.11E-06

LFC: log fold change (the effect size); *P*-value represents the probability that the fold change is equal to 0. ISq = 100% × (*Q* − d*f*)/*Q* represents the percentage of between-variance over total variance; *P*-value–*Q* represents the probability that the variance is coming from within-study only.

### PPAR-encoding mRNAs in total PBMCs of patients with early-onset schizophrenia and of normal controls

**Figure 3:**
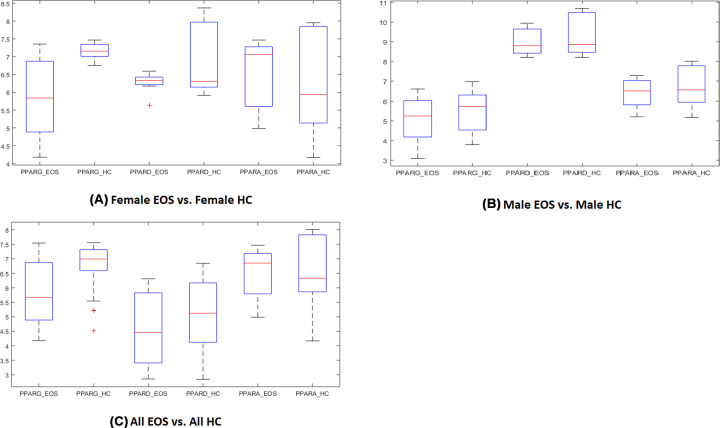
The expression levels of PPAR encoding genes in PNMCs collected from early onset SCZ patients and healthy controls EOS: early-onset schizophrenia group. HC: healthy control group. (**A**) Comparison results from Female EOS vs. Female HC. (**B**) Comparison results from Male EOS vs. Male HC. (**C**) Comparison results from All EOS vs. All HC.

For each of PPAR-encoding genes, the log fold change (LFC) of three comparisons (All EOS vs. All HC; Female EOS vs. female HC; and Male EOS vs. Male HC) were presented in (Figure 3), with detailed statistics are presented in Table 4 and in Supplementary Materials→ Expression Data, Expression Data_Female, and Expression Data_Male. As shown in Table 4, none of the three PPAR encoding genes passed the criteria (LFC≥1 or ≤-1 and *P*-value<0.05) for DEG selection in any of the comparisons. However, in the Female group (Female EOS vs. Female HC), *PPARG* and *PPARD* passed one of the two criteria for DEG selection each. Specifically, *PPARG* demonstrated non-significant but sizeable LFC (−1.30), while *PPARD* presented with a significant decrease in its expression of relatively small magnitude (LFC = −0.55). Therefore, we have to conclude that, as compared with PBMCs of matched controls, PBMCs of early-onset SCZ patients displayed no significant alterations in the expression levels of any of the three PPAR encoding genes. However, the influence of *PPARG* and *PPARD* on the SCZ phenotypes in females may not be excluded.

**Table 4:** The expression levels of PPAR encoding genes in peripheral blood cells

Study design	GeneSymbol	log fold	*P*-value	MeanEOS	StdEOS	MeanHC	StdHC
**All EOS vs. All HC**	PPARG	−0.82	0.22	5.85	1.09	6.67	0.90
	PPARD	−0.54	0.34	4.63	1.33	5.17	1.19
	PPARA	0.02	0.49	6.50	0.86	6.47	1.20
**Female EOS vs. Female HC**	PPARG	−1.30	0.13	5.86	1.13	7.15	0.23
	PPARD	−0.55	0.02	6.31	0.26	6.85	0.97
	PPARA	0.28	0.39	6.57	0.97	6.29	1.40
**Male EOS vs. Male HC**	PPARG	−0.43	0.36	5.08	1.24	5.51	1.13
	PPARD	−0.26	0.35	8.98	0.68	9.25	1.04
	PPARA	−0.26	0.36	6.4	0.74	6.65	1.00

## Discussion

Overwhelming evidence indicates that the signals sent by a family of PPARs participate in the development of brain disorders, presumably through their requirement for balanced regulation of oxidative stress [28]. Indeed, PPARs are all expressed throughout the brain [29], and display certain neuroprotective effects. Moreover, their roles in differentiation, myelination, glutamate toxicity, and Ca^2+^ homeostasis in the brain are also well-described [28]. With that, relative scarcity and inconclusive character of evidence connecting inherited variation in PPAR-encoding loci and psychiatric diseases, including schizophrenia, is surprising.

This study explored the potential association between variation in each of three PPAR-encoding genes (*PPARA, PPARD*, and *PPARG*) and SCZ at different levels, using public large-cohort datasets as well as our own collected gene expression data. The results showed that, in the case of SCZ, the genetic variance and expression changes of PPAR encoding genes do not contribute to schizophrenia significantly.

In a large-cohort GWAS dataset [25], the SNPs that were located in the vicinity of *PPARA* and *PPARD* loci presented with corrected *P*-values>0.39 and >0.15, respectively (see Table 2). When all ascertained variants were taken into account (*N*=9,444,230), one *PPARG* polymorphisms, rs62242085 (bp: 12299345), presented with a corrected *P*-value of 0.035. Nevertheless, even this variant cannot be confirmed as associated with SCZ, because at least 0.6% (*N*=99,040) of all ascertained variants yielded a significance even higher than that of rs62242085. Moreover, no studies reporting any association between this SNP and any diseases were identified. To note, the GWAS samples analyzed here were collected from both European and Asians [25]. Therefore, presented results are representative in terms of human populations, and are consistent with multiple previous studies in their overall conclusion about no allelic association between variation on PPAR encoding genes and SCZ [10,23,24]. Moreover, previously reported findings concerning *PPARD* variant rs2076169 [8] and the *PPARA* variant rs1800206 [9] were not replicated in the large-cohort GWAS datasets employed in the present study (34,241 SCZ cases and 45,604 healthy controls; *P*=0.09 and 0.94, respectively). This may occur due to multiple reasons, including variation in sample size, population sampling regions, and study targets. The samples used in Sun et al.’s study [11] were from the Chinese population (233 SCZ cases; 533 controls). The sample size in Nadalin et al. study [9], where the nicotine dependency among patients with SCZ was investigated, is extremely small (*N*=267).

Mega-analysis of mRNA expression performed in 14 independent case–control datasets covering various regions of the human brain also indicated that none of the three PPAR-encoding loci presented significance in the SCZ versus healthy control comparison (LFC < 0.08; *P*-value > 0.14). These expression profiles were also acquired from different ethnic populations, collected all over the world (see Table 1: Japan; United Kingdom; U.S.A.; China; Brazil). MLR results showed that the population region (country) was a significant factor contributing to the observed variation in expression levels of *PPARA*, but not of *PPARD* and *PPARG* (Table 3). Additionally, brain tissue source was also a significant factor for all the PPAR-encoding genes (*P*<0.0015). However, heterogeneity analysis showed that observed between-studies variance was not significant (*P*-value−*Q* > 0.63; see Table 3), which suggests that the PPARs' expression levels within different human brain regions sampled in SCZ patients from different populations present no significant SCZ-related alterations. For the detailed results of the Mega-analysis and MLR analysis, please refer to the Supplementary Materials→GEO mega-analysis.

PBMC expression data analysis confirmed the GWAS study results and mega-analysis results. According to this analysis, SCZ patients are not different from healthy controls in the expression levels for all three PPAR-encoding genes (Figure 3 and Table 4). However, gene expression of *PPARG* presented an average mean LFC of −1.30 in female early-onset SCZ patients when respective samples were compared with that of the female controls, with significant variance (std = 1.13) paired with non-significant *P*-value (0.13). On the other hand, gene *PPARD* demonstrated a significant change in the expression level of a relatively small magnitude (LFC = −0.55; *P*-value = 0.02). In contrast, the male group (Male EOS vs. Male HC) showed no apparent changes (abs (LFC)<0.43; *P*>0.35) in respective expression levels (Table 4). Thus, our results point toward a necessity for further exploration of the potential roles of *PPARG* and *PPARD* in the development of SCZ in females.

## Conclusions

This study of PPAR-encoding genes expression in PBMCs collected from patients with treatment-naïve early-onset SCZ and from matched controls has revealed an overall lack of disease-associated pattern (see Table 4). As the results from the present study are consistent with a majority of the previous studies [10,23,24], it is tempting to conclude that the chapter on the possible involvement of the variation in human PPAR-encoding in schizophrenia risk should be closed. However, the current study is not free of limitations. In particular, the longitudinal data following the changes in the patterns of PPAR-encoding gene expression throughout a course of schizophrenia, or the patterns collected at a time point preceding the onset of illness are missing. Therefore, it is possible, that some variants in PPAR-encoding loci may still display an association with particular physiological characteristics of SCZ patients [6–9], or their response to the treatment, for example, the treatment-induced weight gain during the administration of antipsychotic medications. Another possibility is that the roles of PPAR encoding genes are gender-specific, as our results pointed at the potential roles of *PPARG* and *PPARD* in the development of SCZ in females.

It is also possible that the variation in genetically and physically separated PPAR-encoding loci should be evaluated together rather than apiece, as a tight cross-talk between the PPAR isoforms, with a positive feedback loop between PPAR-β/δ and PPAR-γ and a negative feedback loop between PPARα and PPAR-β/δ, have been extensively described [28]. This approach would require the development of a systems biology model of PPAR contribution to psychiatric diseases, to be validated in further studies.

## Supplementary Material

Supplementary Table S1

## Data Availability

All data used were included in the manuscript and additional results were available in the Supplementary Material.

## Abbreviations

DEG: differentially expressed gene
FEM: fixed-effect model
GWAS: Genome-wide association study
LFC: log fold change
PBMC: peripheral blood mononuclear cell
PPAR: peroxisome proliferator-activated receptor
REM: random-effects model
SCZ: schizophrenia
